# Glucagon-Like Peptide-1 Receptor Agonists as a Non-surgical Alternative to Bariatric Surgery for Weight Loss: A Review

**DOI:** 10.7759/cureus.100704

**Published:** 2026-01-03

**Authors:** Chuh Steven, Thangwaritorn Skylynn, Haghighat Bardya, Bhalla Megha, Vuong Helen, Duo Lee, Fateh Entabi, Sudhakar Pemminati

**Affiliations:** 1 Department of Biomedical Education, California Health Sciences University College of Osteopathic Medicine, Clovis, USA; 2 Department of Surgery, Adventist Health System, Tulare, USA; 3 Department of Clinical Medicine, California Health Sciences University College of Osteopathic Medicine, Clovis, USA

**Keywords:** bariatric surgery, gastric banding, glucagon-like peptide-1 receptor agonist, liposuction, orexin-a, weight loss and obesity

## Abstract

Obesity is a growing global epidemic, contributing to major health conditions such as cardiovascular disease (CVD), stroke, type 2 diabetes mellitus (T2DM), and cancer. The purpose of this review is to evaluate and compare obesity management strategies, with a focus on the magnitude of weight loss achieved and the adverse effects associated with each intervention. This review was conducted in accordance with the Preferred Reporting Items for Systematic Reviews and Meta-Analyses (PRISMA) guidelines to ensure methodological rigor in study selection and reporting. Bariatric surgery is currently considered the most effective intervention for achieving significant and sustained weight reduction, particularly in patients with severe obesity. Procedures such as Roux-en-Y gastric bypass (RYGB), sleeve gastrectomy (SG), and adjustable gastric banding consistently demonstrate superior weight loss and metabolic improvements compared with nonsurgical therapies. However, surgery carries well-documented risks, such as gastric strictures, anastomotic leaks, thromboembolic events, infections, and the need for reoperations. Long-term challenges include nutritional deficiencies, GI complications, and, in some patients, partial weight regain. Pharmacologic options are increasingly important adjuncts or alternatives to surgery. Novel agents, such as glucagon-like peptide-1 receptor agonists (GLP-1RAs), have gained popularity due to their substantial efficacy among pharmacological options, favorable safety profile, and non-invasive nature. Other agents, including phentermine-topiramate (PHEN-TPM), naltrexone-bupropion, and orlistat, also play roles in individualized treatment plans, although weight loss outcomes are typically more modest than with surgical approaches. In conclusion, a multimodal strategy integrating surgical and medical therapy may provide the most durable results. This approach not only enhances weight loss but also supports long-term maintenance and optimizes management of obesity-related comorbidities.

## Introduction and background

Obesity-associated chronic diseases 

**Figure 1:**
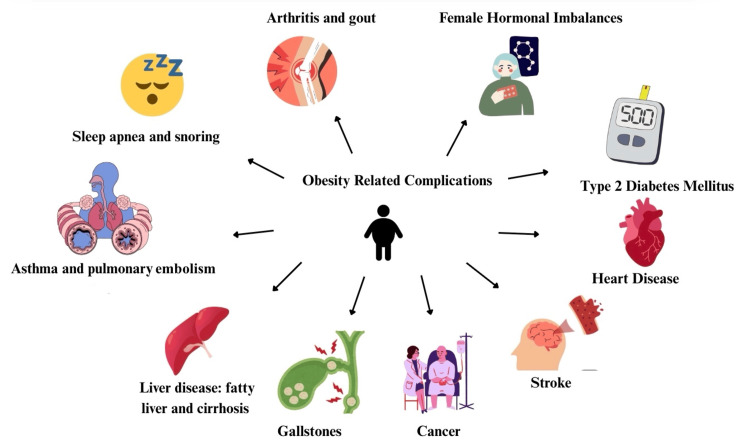
Health complications associated with obesity Image credit: Skylynn Thangwaritorn

Individuals are deemed obese when they have a body mass index (BMI) greater than or equal to 30 kg/m², which is calculated by dividing weight in kilograms by height in meters [1]. Just under the category of obesity is overweight, which is defined as a BMI between 25 and 29.9 kg/m² [1]. We now know the negative health concerns caused by obesity. Some of these health complications include type 2 diabetes mellitus (T2DM), cardiovascular disorders, gallbladder disorders, and breast and endometrial malignancies, as depicted in Figure 1. The United States will have an increased prevalence of obesity, nearly 50%, and overweight, 30%, by 2030, respectively [1]. 

Pathophysiology of obesity

The pathophysiology of obesity is highly complex due to the interplay of genetics, environment, and lifestyle in patients [2]. Genome sequencing has revealed the identification of >300 loci associated with adiposity; notable genes include leptin (LEP) and pro-opiomelanocortin (POMC) [2]. LEP is a hormone, secreted by white adipose tissue, that regulates satiety [2]. LEP accomplishes this by stimulating the downstream POMC neurons in the arcuate nucleus of the hypothalamus. POMC is cleaved to produce alpha-melanocyte-stimulating hormone (α-MSH). α-MSH binds to the melanocortin-4 receptor (MC4R) in the paraventricular nucleus. This activation reduces appetite and increases energy expenditure. LEP also inhibits agouti-related peptide (AgRP) and neuropeptide Y (NPY) neurons. These neurons normally promote feeding and inhibit MC4R. So, LEP suppresses their orexigenic (appetite-stimulating) effects [2]. When the body senses a need for more energy, it triggers the release of NPY to promote increased food intake and decreased energy expenditure [2]. Surprisingly, LEP levels are increased in obese individuals, implying LEP resistance [2]. Like LEP, insulin also provides negative feedback to the brain, leading to decreased food consumption by crossing the blood-brain barrier and acting on the hypothalamus, particularly the arcuate nucleus, and it stimulates POMC neurons and inhibits AgRP/NPY neurons, similar to LEP, promoting anorexigenic effects [2]. Obesity is connected with insulin resistance due to hypersecretion of insulin, leading to greater adipocyte uptake of fatty acids and glucose while simultaneously reducing lipolysis. Despite high circulating insulin, central insulin resistance may develop, blunting its anorexigenic effects. This contributes to persistent hyperphagia and weight gain, creating a vicious cycle [2]. 

Management of obesity

When treating a disorder such as obesity, physicians would use many methods, including lifestyle choices such as diet and exercise, therapies, or invasive last-line procedures such as bariatric surgery. If that doesn't work well enough, some patients are given medications, including semaglutide, tirzepatide, liraglutide, phentermine + topiramate, bupropion + naltrexone, and orlistat. However, due to increased risks and side effects, bariatric surgery isn't usually considered until all other options have been explored for patients looking for rapid weight loss and individuals with a BMI ≥ 40 kg/m² regardless of comorbidities; a BMI ≥ 35 kg/m² with at least one obesity-related condition such as hypertension, T2DM, obstructive sleep apnea, dyslipidemia, or nonalcoholic fatty liver disease; or a BMI ≥ 30 kg/m² considered in select cases, especially in patients with uncontrolled T2DM [3]. Notably, within bariatric surgery, there are also many different options of surgeries, such as the gastric sleeve, Roux-en-Y gastric bypass (RYGB), duodenal switch procedures, and last but not least, the gastric banding surgeries. Specifically, although typically safe, some complications of surgery, such as the gastric sleeve, include inherent risks such as the development of gastric strictures, bleeding along the staple line, and other issues like post-surgery leakage of gastric components, causing life-threatening peritonitis. As is typical with most surgeries, general risks such as deep vein thrombosis and portal vein thrombosis may occur due to long periods of immobilization from rest and recovery. Similarly, the RYGB procedure can cause complications like anastomotic leaks early in the recovery course [4].

**Figure 2:**
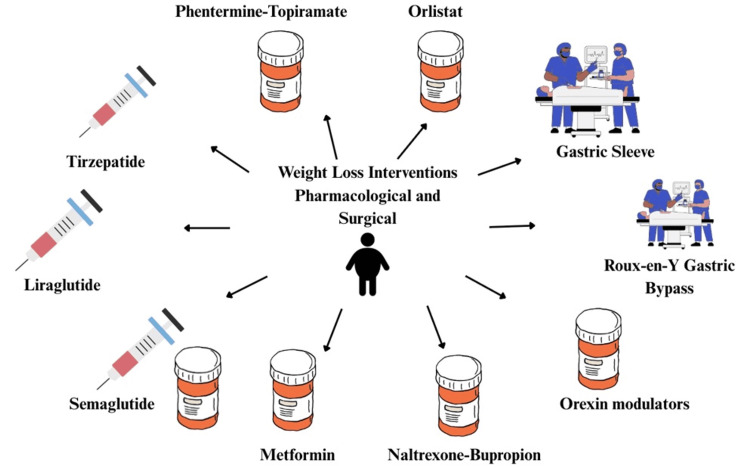
Pharmacological and surgical interventions for the management of obesity Image credit: Skylynn Thangwaritorn

Other issues that may occur include postoperative hemorrhage, Ogilvie syndrome, bowel obstructions, anastomotic adhesions and stricture, gastrogastric fistulas, marginal ulcers, and finally failure of the surgery with a weight regain. Along the same lines, laparoscopic adjustable gastric banding (LAGB) has been shown to have high reoperation rates following the development of erosion of the gastric band or with the regaining of weight in the post-surgery months. More modern innovative procedures like single anastomosis duodenal-ileal bypass with sleeve gastrectomy (SADI-S) are needed to combat these issues. But either way, these procedures still have the risk for chronic diarrhea and malnutrition from the improper absorptive time of food through the GI tract. By the exact same mechanisms, rapid weight loss exacerbates and can cause complications like nutritional deficiencies, GI dysfunction, and imbalances of the metabolic system. Research from meta-analyses has demonstrated the effectiveness of procedures such as one anastomosis gastric bypass (OAGB) with an excess weight loss (EWL) of about 80.9% at 12-24 months post-op, outperforming sleeve gastrectomy (SG) and LAGB as depicted in Figure 2 [5,6]. As noted above, because of such higher postop complications, physicians must do a thorough selection of surgery candidates, meticulous preop counseling, and lifelong postop monitoring to ensure the best outcome possible for patients in terms of safety profiles and long-term success rates [7].

## Review

Methods

We searched through several databases for this review between February 23, 2025, and April 21, 2025. The last search date was November 4, 2025. We mainly used PubMed/Medical Literature Analysis and Retrieval System Online (MEDLINE), Google Scholar, Excerpta Medica database (EMBASE), Web of Science, and the Cochrane Library. We tried out a mix of search terms. Some of these included terms like "Glucagon-like peptide agonists," "Semaglutide," "Obesity," "Semaglutide safety," "Semaglutide without diabetes," "Semaglutide non-diabetic," "Bariatric surgery," "Exercise weight loss," "Metformin AND weight loss," "Metformin versus bariatric surgery," "GLP-1RA adverse effects," "Tirzepatide weight loss," and "Obesity United States." We set the time frame for articles mostly from 2010 to 2025, since the focus was on getting data on more recent advances.

The database search produced many results, well over 36,366 entries. PubMed contributed the most targeted clinical studies, with 638 results for "Glucagon-like peptide agonists," 101 for "Semaglutide, Obesity," and 358 for "glucagon-like peptide-1 receptor agonist (GLP-1RA) adverse effects." Only articles from peer-reviewed journals with full-text access were kept, narrowing the scope. After this initial filtering, studies were manually checked for actual relevance.

Inclusion criteria were studies on GLP-1 receptor agonists, bariatric surgery, and weight-loss drugs used for comparison. Established treatments (semaglutide, liraglutide, tirzepatide, and metformin) and newer agents being studied were included in the criteria. Preference was given to work that described drug mechanisms, weight-loss outcomes, side-effect profiles, and longer-term results, especially in studies that compared medications directly with bariatric surgery. 

We excluded studies that were not available in English, were not in the given time frame, lacked clear outcomes, duplicated studies, or did not have available full-text articles.

**Figure 3:**
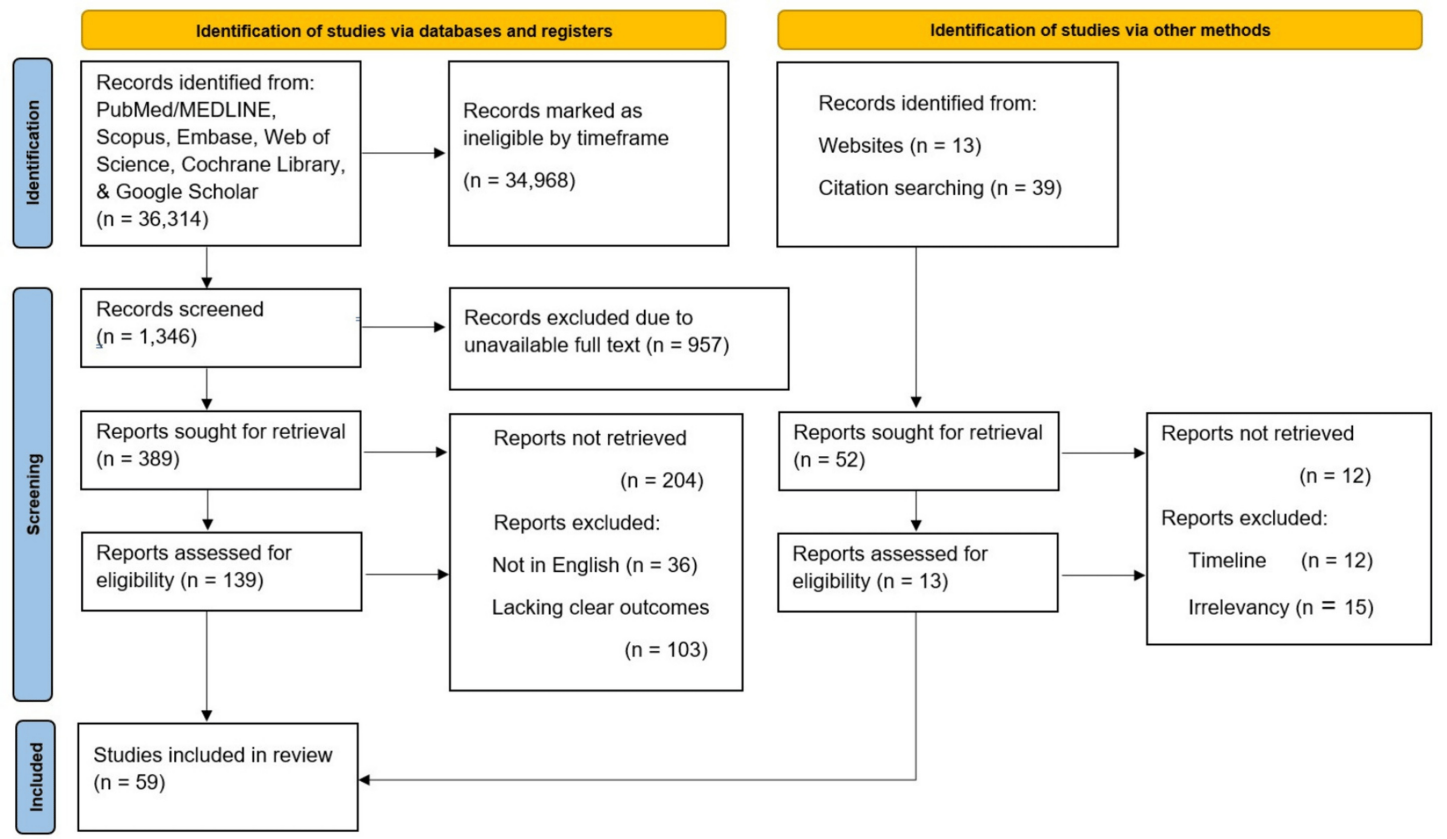
Preferred Reporting Items for Systematic Reviews and Meta-Analyses (PRISMA) flow chart of inclusion and exclusion criteria MEDLINE: Medical Literature Analysis and Retrieval System Online

A second manual review was done to catch studies that might have been overlooked in the electronic search and to screen out irrelevant ones. For emerging treatments, only articles from 2010 onward were included. Ultimately, 59 studies met our stringent criteria and were included in the review. Preferred Reporting Items for Systematic Reviews and Meta-Analyses (PRISMA) recommendations were followed, as Figure 3 displays [8].

The role of GLP-1RA in obesity

Glucagon plays a significant role in regulating blood glucose by preventing glucose levels from dropping too low through glycogenolysis. GLP-1 acts against glucagon by simultaneously facilitating insulin secretion and reducing glucagon secretion. This process results in weight loss through the inhibition of stomach emptying and facilitation of satiety. GLP-1 further influences gastric motility by increasing pyloric sphincter tone and frequency of pyloric pressure waves, thereby slowing the transfer of gastric contents into the small intestine for absorption [9].

It is important to note that GLP-1 does not compete with glucagon for the same receptor binding. Instead, GLP-1 specifically binds to GLP-1R on beta cells in the pancreas, while glucagon binds G protein-coupled receptors (GPCRs) located in a variety of organs, including the liver, brain, heart, and kidneys. GLP-1 also has a short duration of action due to its degradation by dipeptidyl peptidase-4 (DPP-4). Extending the half-life of GLP-1 was paramount in the development of GLP-1RA, which can resist DPP-4's ability to degrade. Variants of GLP-1RA have different pharmacokinetic profiles that result in varying administration and half-life, but all result in weight loss [10]. 

Liraglutide

Liraglutide is a GLP-1RA that directly results in the adenylate cyclase pathway, leading to increased cAMP and subsequent activation of protein kinase A (PKA) that simultaneously closes adenosine triphosphate (ATP)-dependent K+ channels and opens Ca2+ channels, which facilitates glucose-dependent insulin release. Central effects include liraglutide binding to GLP-1R in the hypothalamus that increases satiety, leading to reduced intake of food [11].

A randomized, placebo-controlled trial with non-diabetic adults who had a BMI between 32 and 43 had participants undergo eight weeks of a low-calorie diet. Afterward, they were randomly assigned to one of four different groups: placebo, exercise (each participant was assigned to an instructor (who had a bachelor’s or master’s degree in exercise physiology) who planned and monitored the individualized programs. After an initial six-week ramp-up phase, participants were encouraged to attend supervised group exercise sessions (which involved 30 minutes of vigorous-intensity, interval-based indoor cycling and 15 minutes of circuit training) two times per week and to perform moderate-to-vigorous-intensity exercise individually (which mostly involved outdoor or indoor cycling, running, or brisk walking) two times per week. Heart-rate monitors were worn at all exercise sessions to determine whether the requirement regarding weekly time spent at moderate or vigorous intensity was met, liraglutide or exercise, and liraglutide for a one-year duration. Patients in all active treatment groups experienced weight loss compared to placebo. The most effective weight loss was observed in the combination of exercise and liraglutide, leading to a 9.5 kg (95% CI, −13.1 to −5.9; P<0.001) loss compared to just the exercise group, 6.8 kg (95% CI, −10.4 to −3.1; P<0.001) with liraglutide alone, 4.1 kg (95% confidence interval (CI), -7.8 to -0.4; P=0.03) with exercise alone, and minimal change with placebo. The combination of exercise and liraglutide, therefore, provided the most robust weight reduction (difference, −5.4 kg; 95% CI, −9.0 to −1.7; P=0.004), but not liraglutide (−2.7 kg; 95% CI, −6.3 to 0.8; P=0.13), and a strategy combining exercise and liraglutide therapy improved healthy weight loss maintenance more than either treatment alone [12].

Semaglutide

Semaglutide is a long-acting GLP-1RA with a molecular structure closely resembling native human GLP-1. Structural modifications reduce renal clearance and confer resistance to degradation by DPP-4, enabling sustained therapeutic concentrations. Semaglutide exerts effects across multiple organs: in the brain, it enhances satiety; in the pancreas, it promotes glucose-dependent insulin secretion and suppresses glucagon release; and in the stomach, it delays gastric emptying. Collectively, these mechanisms result in significant weight reduction [13-15].

The efficacy of semaglutide has been demonstrated in the global STEP (Semaglutide Treatment Effect in People with Obesity) phase 3 clinical trial program [16]: STEP 1 was a 68-week randomized, placebo-controlled trial that assessed weight management in 1961 adults with a BMI of ≥30 kg/m² (or ≥27 kg/m² with ≥1 weight-related comorbidity) and without type 2 diabetes [17].

Participants were assigned to once-weekly subcutaneous semaglutide 2.4 mg vs. placebo in adults with obesity, showing a mean weight loss of -12.5 kg (14.9% body weight reduction, 95% CI -13.4 to -11.5) compared with placebo. STEP 2 compared semaglutide 1.0 mg vs. 2.4 mg vs. placebo in adults with type 2 diabetes, highlighting the superior efficacy of the 2.4 mg dose. STEP 3 confirmed the benefits of semaglutide 2.4 mg plus intensive behavioral therapy compared with placebo. STEP 3, with the provision of meal replacement products (for the first eight weeks) and intensive behavioral therapy (IBT, 30 counseling sessions over 68 weeks), increased weight loss in the placebo group to 5.7% at week 68. The loss approached 8% at week 24, before participants regained weight in the remainder of the study. Study investigators had thought that the addition of semaglutide to meal replacements and IBT might boost total weight loss to 18% to 20% of initial weight. However, this combined intervention produced a 16% loss at week 68, only one percentage point greater than that observed in STEP 1 [18].

STEP 4 examined the effect of continuing versus withdrawing semaglutide treatment after 20 weeks of initial therapy in adults with a BMI ≥30 kg/m² (or ≥27 kg/m² with ≥1 weight-related comorbidity) and without diabetes. One group continued semaglutide 2.4 mg + lifestyle intervention (535 subjects), and the other group switched to placebo + lifestyle intervention (268 subjects). After 20 weeks, 803 participants achieved the 2.4 mg/week maintenance dose, lost an average of 10.6% of their initial weight, and agreed to continue in a 48-week randomized trial in which they were assigned in double-blind fashion to continued semaglutide or switched to placebo. Those who remained on semaglutide lost 7.9 percentage points of their randomization weight from weeks 20 to 68, whereas those switched to placebo gained 6.9 percentage points (estimated treatment differences = -14.8; 95% CI=-16.0 to -13.5 percentage points; p<0.001). As measured from the start of the run-in period, the two groups achieved mean cumulative weight losses at week 68 of 17.4% and 5.7%, respectively. Overall, semaglutide 2.4 mg once weekly has shown robust and sustained weight-loss effects, with efficacy superior to lifestyle interventions and lower doses, particularly when maintained for at least 12 months. Semaglutide has demonstrated the largest weight loss among GLP-1RA-only obesity medications to date, with reductions of approximately 15% of initial weight at 68 weeks, accompanied by improvements in cardiovascular risk factors and physical functioning. The approval of this medication provides patients with greater options for weight management [19]. 

Tirzepatide

Furthermore, tirzepatide is a dual agonist medication for both the GLP-1RA and the glucose-dependent insulinotropic polypeptide (GIP) receptor and has been shown in some studies to be more effective in weight loss, up to 22.5%, compared with GLP-1RAs [20]. Specifically, GIP during hyperglycemic levels of glucose-dependent insulinotropic peptide will induce insulin to be released, which in turn lowers glucagon through feedback mechanisms, while during euglycemic or hypoglycemic conditions, GIP will cause glucagon levels to increase. Within the adipose tissue, there are a myriad of GIP receptors in which GIP triggers the postprandial lipid buffering abilities of the white adipose tissue as well as its insulin sensitivity, which may hinder the deposition of ectopic fat.

Additionally, the GIP agonism may act on key feeding centers in the brain to result in a GLP-1-induced decrease in food consumption. Head-to-head trials comparing tirzepatide to semaglutide demonstrated superior weight reduction across escalating doses: treatment differences of -1.9 kg, -3.6 kg, and -5.5 kg for tirzepatide 5 mg, 10 mg, and 15 mg, respectively [21]. In the SURMOUNT-1 trial (Efficacy and Safety of Tirzepatide Once Weekly Versus Placebo in Participants Who are either Obese or Overweight with Weight-Related Comorbidities), tirzepatide at 5 mg led to a 16.1 kg (35.5 lb) weight reduction across 72 weeks and increased weight loss at greater dosages [22]. Tirzepatide is currently the most effective dual GIP/GLP-1-based therapy for weight loss, achieving reductions of over 20% in many subjects. However, long-term real-world durability remains under investigation.

Additional Benefits of GLP-1RAs

Beyond weight loss, GLP-1RAs may provide cardiometabolic and anti-inflammatory benefits. Preclinical studies suggest immunomodulation: reduction in TNF-α release and attenuation of systemic inflammation, with protective effects in sepsis, lung injury, and hypothermia models [23]; clinical studies suggest cardiovascular protection: reduction of cardiac fibrosis, aortic wall thickness, and blood pressure through downregulation of NADPH oxidase (NOX4), TGF-β, and adhesion molecules, with enhanced nitric oxide synthase activity [24]; and hepatic protection: indirect benefits on steatosis and fibrosis through reduced postprandial lipoprotein secretion and modulation of systemic inflammation, potentially mitigating metabolic-associated fatty liver disease [25,26].

Adverse Effects and Safety Concerns

Like all GLP-1RAs, semaglutide, liraglutide, and tirzepatide are associated with GI adverse effects, including nausea, vomiting, diarrhea, and constipation. Additional risks include rare but serious events like cholelithiasis, pancreatitis, acute kidney injury, angioedema, diabetic retinopathy, and hypoglycemia (especially in combination with insulin or sulfonylureas) [27]. 

Contraindications: Semaglutide is contraindicated in pregnancy and in patients with a history of medullary thyroid carcinoma or multiple endocrine neoplasia syndrome type 2. Emerging safety signals: observational data suggest an association between semaglutide and non-arteritic anterior ischemic optic neuropathy (NAION). Observational data suggest an increased relative risk of NAION among semaglutide users compared to controls in diabetic cohorts, though causality is not established, and absolute incidence remains low. This is hypothesized to result from sympathetic nervous system-mediated alterations in optic nerve head perfusion [28]. The findings are summarized in Table 1.

**Table 1:** GLP-1RAs in the management of obesity GLP-1RAs: glucagon-like peptide-1 receptor agonists; LDL: low-density lipoprotein; STEP: Semaglutide Treatment Effect in People with Obesity; HbA1c: hemoglobin A1c

Medication, Author, Year, & Country	Study Population	Mechanism of Action	Risk	Benefits
Liraglutide, Tamayo-Trujillo et al, 2024, Ecuador [11]	Review	GLP-1R (increased insulin and greater lipid metabolism). Hypothalamic GLP-1R (satiety) delays gastric emptying.	Liraglutide-only group reported 37% with gastrointestinal issues (nausea, vomiting, and diarrhea). No specific percentages for the following, only greater incidences of cholelithiasis and palpitations.	The liraglutide-only group reported body fat mass loss of 6%, decreased HbA1c of 0.18%, and decreased insulin resistance by 29%.
Semaglutide, Chao et al, 2023, United States [19]	N=1961	Enhances insulin secretion, suppresses glucagon release, delays gastric emptying, and acts on the hypothalamus to increase satiety.	The semaglutide 2.4 mg group reported nausea, diarrhea, constipation, cholelithiasis, and acute pancreatitis.	No exact quantification for the following benefits. Lower systolic and diastolic blood pressure, hemoglobin A1c, fasting blood glucose, total cholesterol, LDL cholesterol, and triglycerides.
Tirzepatide, Boer et al, 2023, United States [20]	N=1879	Acts on key feeding centers in the brain to decrease food intake, shifts fat from ectopic (visceral, liver) sites to healthier abdominal subcutaneous adipose tissue (aSAT), leading to a more balanced fat profile, enhances postprandial lipid buffering abilities of white adipose, and increases sensitivity to insulin in white adipose tissue.	Nausea (17.4% - 19.2%), diarrhea (13.2% -16.4%), constipation (6.8% - 4.5%), acute pancreatitis (0.4%), cholelithiasis (0.9%), diabetic retinopathy (0.4%), hypersensitivity reactions (2.8%) [21].	Improvement of glycated hemoglobin levels >8.5 (37.3%). No exact quantification for improved fasting glucose and lower LDL cholesterol, along with improvements in systolic and diastolic blood pressures.

Other pharmacological agents for obesity management 

Metformin

Although not FDA-approved for weight loss, metformin is used off-label for those who are overweight or obese and have type 2 diabetes or prediabetes. A first-line treatment for type 2 diabetes, metformin decreases hepatic glucose production and improves insulin sensitivity by reducing the need for excessive insulin production. The mechanisms for weight loss have been hypothesized to be due to the regulation of hypothalamic appetite regulatory centers, enhancement of the gut microbiome, and reversal of the sequelae of cell aging. Metformin decreases hepatic glucose production and modulates mitochondrial energetics and redox potential by inhibiting complex I of the electron transport chain, therefore reducing ATP production. Furthermore, metformin activates AMP-activated protein kinase (AMPK), which inhibits hepatic gluconeogenesis, increases insulin sensitivity, mildly suppresses appetite, and increases secretion of GLP-1 in addition to the anorectic hormone peptide YY [29].

In the diabetes prevention program, about 29% of patients on metformin versus 13% on placebo lost at least 5% of baseline weight at one year, and about a quarter of metformin-treated patients maintained ≥5% loss at two years [30]. By comparison, semaglutide and tirzepatide achieve far greater reductions of ~16% and ~20-25%, respectively [31,21].

Lorcaserin

Lorcaserin is a selective serotonin 2C receptor agonist shown to induce weight loss in overweight individuals. 5-HT 2C receptors on the POMC neurons in the arcuate nucleus cause the release of α-MSH that may modulate MC4Rs within the paraventricular nucleus, acting to suppress appetite [32]. In clinical studies, 73.9% of lorcaserin-treated participants achieved ≥ 5% weight loss in 24 weeks, compared with 57.4% in the placebo group, with additional improvements to cardiometabolic risk factors. However, by week 52, there was no statistical difference in weight loss in both groups. Other adverse effects included headaches, fatigue, constipation, and dry mouth [33]. Importantly, lorcaserin was withdrawn from the U.S. market in 2020 due to cancer risk, and it is no longer recommended for weight management [34].

Orexin

Orexin is also known as hypocretin and is a neuropeptide originating from the hypothalamus that has been shown to affect pathways involved in arousal, wakefulness, and appetite. Prior research has noted the role of orexin modulators in appetite suppressants, in which the same principles might affect weight loss regulation. Rodent research populations have shown that these two forms of orexin, orexin-A and orexin-B, stimulate food intake and appetite. Because orexin receptors have been found in the hypothalamus but also in the pancreas and the gut, it can be surmised that orexins may play a similar part within humans. Within the obese population with BMIs ranging from 19.8 to 59 kg/m², plasma orexin-A levels were found to correlate negatively with BMI, while LEP had a positive correlation with BMI [35]. Following bariatric surgery, patients demonstrate increased orexin levels on par with non-obese individuals and improved lipid panel and fasting glucose data points, maintained over 12 months. Most data are based on animal data, and human data remain preliminary [36].

Phentermine/Topiramate

A sympathomimetic drug, phentermine, stimulates norepinephrine via beta-adrenergic receptors in the hypothalamus, and it is used for various medical conditions, including obesity [37]. In 2012, the FDA approved phentermine and topiramate extended release (PHEN/TPM ER) for the treatment of obesity, especially for patients with a BMI over 30 or those with a BMI over 27 and obesity-related comorbidities such as type 2 diabetes, hypertension, and hyperlipidemia [37,38]. While PHEN is a central nervous system stimulant that increases the release of norepinephrine and epinephrine, TPM is an antiseizure and anti-migraine medication that acts on the high-voltage-activated calcium channels and voltage-gated sodium channels by augmenting GABA-A receptors. PHEN alone has been used as a short-term treatment of obesity with lifestyle modifications; however, the addition of TPM has been shown to augment the suppression of appetite and enhance satiety for weight loss [37].

The OB-202/DM-230 and CONQUER trials demonstrated that PHEN/TPM ER achieved significantly greater weight loss than placebo: Weight change: -9.4% with PHEN/TPM ER vs. -2.6% with placebo, ≥5% weight loss achieved: 65% of PHEN/TPM ER patients vs. 24% of placebo, ≥10% weight loss achieved: 37% vs. 9% [38]. PHEN/TPM ER also improved glycemic control, with HbA1c reductions of 1.6% vs. 1.2% for placebo, and reduced the need for additional antidiabetic medications. 

The common adverse events associated with PHEN/TPM ER were paresthesia, constipation, and insomnia, but only mild events were observed in the study [38]. Moreover, the discontinuation of the medication due to these adverse events was also rare; in fact, only one case of such an adverse event was seen [38]. About 58 hypoglycemic events were reported; these were mild to moderate and occurred due to concurrent use of antidiabetic medications [36]. This side effect is clinically significant, suggesting PHEN/TPM ER may be an effective approach to glycemic control in type 2 diabetes, potentially reducing the need for multiple antidiabetic medications [38].

The following study examined the relationship between food cravings and weight loss [39]. This was a 12-week-long randomized, double-blind, and placebo-controlled study. The sample size was 96 adults with a BMI of 35 to 50, and the subjects were divided into the placebo or PHEN group [39]. Nonetheless, all the subjects were provided Medifast meal replacements and were instructed to follow the Take Shape for Life Optimal Weight 5 & 1 Plan [39]. The subjects were also provided with lifestyle coaching through the Habits of Health Program for weight loss. Telephone-based lifestyle coaching was utilized. The Food Craving Inventory and the General Food Cravings State and Trait Questionnaires were used to assess food cravings among the subjects. From the total of 96 subjects, 87 subjects were provided with Medifast meal replacements and randomized into either the placebo or PHEN group to evaluate food cravings and weight loss changes [39]. No significant differences were noted between the placebo and treatment group, and these differences included mean weight, height, BMI, body fat percentage, and food cravings.

The participants were predominantly female (82%) and Caucasian individuals (60%) [39]. At the 12-week mark, about 8.8% weight loss was observed in the placebo group, while 12.1% was observed in the PHEN group [39]. There was a significant reduction in the percentage of fat loss in the PHEN group (10.3%) vs. the placebo group (8.2%) with no significant difference in lean body mass between the two groups (3.8% vs. 2.5%) [39]. Therefore, the PHEN group had a higher weight loss reduction than the placebo group, and this coincided with the data on food cravings [39]. Overall, food cravings decreased in both groups, but the PHEN group had a significant reduction in cravings for fats and sweets compared to the placebo group [39]. Therefore, the data suggest that PHEN, in addition to a lifestyle and dietary intervention, is more effective in reducing food cravings than just the lifestyle and dietary intervention alone [39]. This is crucial because food cravings for fats and sweets are highly associated with obesity, diet inconsistency, and overall eating behaviors [39].

Lastly, the following study examined the long-term use of PHEN and its effects on weight loss as well as cardiovascular disease (CVD) or death [40]. The data were obtained from electronic health records, and about 13,972 adults were identified who had first filled the prescription for PHEN in 2010 to 2015 [40]. Utilizing multivariable linear models and Cox proportional hazards models, the risk of CVD or death was assessed while being on PHEN for 36 months [40]. The results showed that patients on longer-term use of PHEN achieved greater weight loss. In fact, about 7.4% of weight loss was observed among patients who were on PHEN for more than 12 months [40]. In addition, the composite CVD, or death outcome, was 0.3%, or extremely rare, with no significance between the two groups in terms of hazard ratios [40]. This study supported the efficacy and safety of PHEN in long-term use for weight loss.

Naltrexone-Bupropion

A combination medication, naltrexone-bupropion extended-release, is used for weight management in overweight or obese individuals, and its primary role is to target both appetite and cravings [41]. Naltrexone is a mu-opioid receptor antagonist that reduces the reward associated with eating, while bupropion is a norepinephrine-dopamine reuptake inhibitor that helps control food cravings and promote satiety [41].

Common side effects include headaches, dizziness, nausea, constipation, insomnia, and increased risk of seizures in individuals with eating disorders like bulimia nervosa or other medical conditions like alcohol use disorder and uncontrolled hypertension [42]. It can also cause mood changes such as suicidal thoughts or behavior. These medications can also increase blood pressure; therefore, continued monitoring of blood pressure is required, especially in patients with existing hypertension or cardiovascular conditions [42]. Despite these potential risks, this drug is still considered effective for long-term weight management when carefully monitored under the supervision of a physician [41]. 

Clinical evidence supports modest but significant weight loss with this therapy. A systematic review of four randomized controlled trials (N=3955) found that 53% of patients achieved ≥5% weight loss and 29% achieved ≥10% weight loss at week 56, compared with 21% and 13% in placebo groups, respectively. Another systematic review (N=4536) reported similar outcomes with lower doses (16/360 mg daily), with 38% of patients achieving ≥5% weight loss vs. 17% with placebo, though high dropout rates (up to 45%) and adverse event-related discontinuations (~25%) were noted. Nausea and constipation were the most common reasons for withdrawal [43].

Another systematic review and meta-analysis were done from five databases, and it included twenty-five studies with 22,165 participants [44]. This analysis from randomized controlled trials showed that body weight and waist circumference had significantly reduced with the treatment of naltrexone/bupropion or bupropion only vs. placebo [44]. The findings showed that weight decreased by 3.67 kg with bupropion, and waist circumference decreased by 2.98 cm with bupropion in comparison to the placebo group [44]. The analysis also showed that these weight loss and waist circumference changes were maintained for at least 26 weeks compared to the control group [44]. Furthermore, these changes were significantly higher with the combination of naltrexone and bupropion medication than with just bupropion alone [44]. Thus, there is evidence of a synergistic effect of this combined medication causing more benefit in terms of weight loss and waist circumference reduction in patients [44]. This greater weight loss is perhaps due to naltrexone/bupropion-based endorphin-mediated autoinhibition of hypothalamic POMC neurons [44]. Overall, this medication could be a great option for patients struggling with obesity, as it has shown long-term weight loss effects in patients with lifestyle modifications.

Orlistat

Orlistat, a gastric and pancreatic lipase enzyme inhibitor, is an anti-obesity medication that suppresses the action of enzymes that are responsible for dietary fat hydrolysis within the GI tract. It suppresses 30% of dietary fat absorption into the bloodstream, resulting in reduced fat caloric consumption [45,46]. This drug is used in combination with lifestyle modifications to help patients who are obese or overweight [45,46]. In a one-year randomized trial of 3,132 obese adults following a hypocaloric diet, patients treated with orlistat achieved 9.2% mean weight loss compared with 5.8% in the placebo group. Additionally, significant reductions in serum lipids were observed, including decreases in total cholesterol (-1.98%) and low-density lipoprotein (LDL) cholesterol (-4.23%) compared to increases in the placebo group (+4.6% and +4.7%) [45,46]. The common adverse effects associated with this medication include oily spotting, flatulence, diarrhea, and frequent bowel movements [45,46]. These side effects result from the undigested fat in the GI tract, but these side effects are mitigated by adhering to a low-fat diet. Severe adverse side effects include liver injury or kidney stones, as well as malabsorption of fat-soluble vitamins (K, A, D, E), which can be reversed with vitamin supplementation. Orlistat is generally well tolerated and has demonstrated efficacy in supporting weight loss, but continued monitoring is required to ensure safety and therapeutic benefit [45,46]. The findings are summarized in Table 2.

**Table 2:** Non-GLP-1RA in the management of obesity ETC: electron transport chain; AMP: activated protein kinase; YY: tyrosine tyrosine; GLP-1RA: glucagon-like peptide-1 receptor agonist

Medication, Author, Year, Country	Study Population	Mechanism of Action	Benefits	Risk
Metformin, Yerevanian, 2019, United States [29]	N=3,234	Inhibits complex I of the ETC, reducing ATP production. Activates AMP-activated protein kinase, which inhibits hepatic lipogenesis and gluconeogenesis, and is an appetite suppressant. Increases secretion of anorectic hormone peptide YY.	5.8% to 8.2% of weight loss over the 2-15 years compared to baseline	Gastrointestinal side effects are common: nausea/vomiting (20-25%), diarrhea (15-20%), abdominal pain (5.3%), and constipation (1.1%). Less common adverse effects are hypoglycemia, allergic reactions, and hepatobiliary disorders.
Lorcaserin, Tronieri et al., 2017, United States [33]	N=137	A selective serotonin 2C receptor agonist releases alpha melanocortin-stimulating hormone from the pro-opiomelanocortin neurons to act on melanocortin-4 receptors in the paraventricular nucleus, which suppresses appetite.	73.9% of the lorcaserin cohort maintained greater than 5% weight loss versus only 57.4% of placebo group participants at the 24-week mark	Headache (17.4%), fatigue (15.9%), constipation (14.5%), dry mouth (10.1%), sialolithiasis (1.5%), cancer (8.6%).
Phentermine and Topiramate, Timothy et al., 2014, United States [38]	N=4,123	Topiramate: Enhances satiety, Phentermine: Stimulates norepinephrine release via beta-adrenergic receptors in the hypothalamus, and suppresses appetite.	5–10% body weight reduction over 56 weeks, up to 12.1% weight loss with phentermine + meal replacements	Dry mouth (19-21%), constipation (16-17%), tachycardia (2%), mood disturbances (4-8%), anxiety (4-8%), kidney stones (<1%), sleep disorders (9-11%), irritability (4%).
Orlistat, Heck et al., 2000, United States [45]	N=29,018	Lipase inhibitors	5-10% body weight loss	Oily spotting (27%), flatulence (24%), diarrhea (5.3%), and frequent bowel movements (10.8%). More serious side effects, such as liver injury (rare) or kidney stones (rare), and malabsorption of fat-soluble vitamins (A, D, E, and K).

Surgical management of obesity

Gastric Sleeve Surgery

SG is widely regarded as an effective and relatively safe bariatric procedure for sustained weight loss [47]. Nonetheless, perioperative and long-term complications may occur. Common risks include gastric stricture, which can obstruct food passage, and staple-line bleeding, which may necessitate transfusion or reoperation. Less frequently, leakage of gastric contents can cause peritonitis, a life-threatening condition requiring urgent surgical repair. Patients are also predisposed to thromboembolic complications such as deep vein thrombosis and portal vein thrombosis, the latter potentially impairing liver function and requiring anticoagulation [48]. Lastly, fewer than one in 50 will experience infection around the incision site [49]. 

Single Anastomosis Duodenal-Ileal Bypass With Sleeve Gastrectomy (SADI-S)

SADI-S consistently yields low complication rates when performed at high-volume, experienced bariatric centers, reinforcing its safety profile and procedural reliability. In one series with a total of 121 patients, only 1.7% required reoperation, and 3.3% developed late complications, most commonly malnutrition and chronic diarrhea [48].

Roux-en-Y Gastric Bypass

RYGB is a common bariatric surgical procedure that can have anastomotic leaks, and postoperative hemorrhages are the main early risk. This involves bleeding from the surgical site or staple lines. Small bowel obstruction may also arise in the early period. This is often due to internal hernias or adhesions, which lead to bowel blockage requiring quick surgical intervention. Late complications can include gastrojejunostomy anastomotic stricture, where scarring at the surgical junction causes narrowing and impairs the passage of food. Additionally, marginal ulceration can form around the surgical connection, and these can lead to perforation, bleeding, and pain. A rare but major complication of gastrogastric fistulas may also occur. This involved the formation of a connection between the new stomach pouch and the remnant stomach. This can lead to gastric reflux or infection. Weight regain is also a long-term concern, as some patients may regain lost weight due to physiological or behavioral factors, despite the initial success of the procedure [4,50].

Gastric Banding

LAGB revealed an 84.8% overall complication rate. These included 17.7% of band erosions and 11.5% of weight regained after the procedure. Additionally, 78.5% of patients required reoperation, with 72.7% having to undergo band removal. Long-term success rate is limited, with only 48.7% achieving durable weight loss and a mean EWL of 47.1% in this study [51].

Biliopancreatic Diversion With Duodenal Switch (BPD/DS)

In a study, a total of 116 patients (83.6% female) underwent BPD/DS with a mean initial BMI of 47 ± 6.5 kg/m². Of these, 68% of the procedures were performed with an open technique and 32% laparoscopically. The majority (76.7%) of patients had LAGB before BPD/DS. BPD/DS is associated with substantial weight reduction, with an average BMI reduction of 78% at five years, and 92.8% of patients achieving partial or complete diabetes remission. However, reoperation was required in 29.3% of cases due to reflux, inadequate weight loss, or malnutrition, highlighting the need for careful patient selection and long-term nutritional monitoring [52].

Intragastric Balloons (IGBs)

IGBs are a temporary, endoscopic intervention for weight loss but carry risks if improperly managed. A study reported 22 cases of gastric perforation, often related to prior surgeries or delayed balloon removal, and 12 cases of bowel obstruction secondary to balloon deflation. Extended implantation increases risks due to material degradation, underscoring the importance of strict adherence to removal protocols [53].

Liposuction

Liposuction is a cosmetic body-contouring procedure that removes subcutaneous fat through suction, ultrasound, or laser-assisted lipectomy. It is best suited for patients within 20-30% of ideal body weight with good skin elasticity [54]. A systematic review of 29,368 patients found an overall complication rate of 2.62% [54]. Reported complications include contour irregularity (2.35%), hyperpigmentation (1.49%), and seroma (0.65%). Severe but rare risks include venous thromboembolism (0.017%) and anesthesia-related toxicity (0.016%). Patient factors such as uncontrolled diabetes, CVD, and smoking increase surgical risks [53]. Although generally safe, liposuction is not a treatment for obesity but rather a contouring technique, and proper preoperative counseling is essential [54,55].

Comparative Outcomes of Bariatric Surgical Procedures

This review synthesizes the effectiveness and safety profiles of commonly performed bariatric surgeries by comparing EWL and reoperation rates. 

One-anastomosis gastric bypass (OAGB): OAGB demonstrated the highest weighted mean EWL at 80.9% (range: 70-84%) with relatively low reoperation rates (2-14%), suggesting it is among the most effective and safe procedures [6,56]. EWL is not the same as total body weight loss (TBWL).

Duodenal switch (DS): DS achieved a mean EWL of 75.2%, though reoperation rates varied widely (3-37%), reflecting potential for higher long-term complications despite excellent weight loss.

Biliopancreatic diversion (BPD): BPD showed a mean EWL of 71.5%, though limited data were available on reoperation rates, making long-term safety assessment more difficult.

Roux-en-Y gastric bypass (RYGB): RYGB produced a mean EWL of 55.4% (range: 27-69%), with reoperation rates ranging from 8 to 64%. While effective, variability in outcomes and relatively higher revision rates highlight the importance of patient selection and surgical expertise.

Laparoscopic sleeve gastrectomy (LSG): LSG achieved a median (range) %EWL of 43.5% (2.1%-109.2%) after LSG, and the overall reoperation rate was 15.7% for LSG and 18.5% for LRYGB (P=0.57) [57].

Gastroplasty: This procedure showed a mean EWL of 50.9% with reoperation rates of 10-40%, indicating modest efficacy with notable risk for surgical revision.

Laparoscopic adjustable gastric banding (LAGB): LAGB was the least effective, with a mean EWL of 45.9% and the widest reoperation range (8-78%), reflecting poor durability and high variability in outcomes.

Overall, OAGB showed the highest mean weight loss with relatively low reoperation rates in available studies [7]. Lastly, while modern bariatric procedures have numerous studies demonstrating their short-term safety, there are fewer studies that tackle beyond five years, and there is a continuing problem with the lack of reporting of the complications in a standardized manner. This makes it difficult to firmly establish the rates of major adverse effects [58]. The findings are summarized in Table 3.

**Table 3:** Surgical management of obesity DVT: deep vein thrombosis; GERD: gastroesophageal reflux disease

Surgery, Author, Year, Country	Mechanism of Action	Benefits	Risk
Gastric Sleeve, Brajcich BC, 2020, Arterburn, D, 2020, United States [49,58]	Restrictive bariatric procedure where ~80% of the stomach is removed, leaving a narrow "sleeve" to limit food intake.	Effective for significant weight loss (~43–55% excess weight loss). No intestinal rerouting; lower malabsorption risk. Lower risk of dumping syndrome than Roux-en-Y gastric bypass. Preserves the pylorus, reducing bile reflux.	Typical early complication rates: leak, 1–3%, bleeding, 1–2%, and stricture, 0.5–2%. Thromboembolic events are uncommon; portal vein thrombosis is rare but reported. Rates vary by center experience and perioperative protocols.
Roux-en-Y Gastric Bypass (RYGB), Aminian A, 2018, Griffith P, 2012, United States [50,4]	Combined restrictive and malabsorptive procedures, where a small gastric pouch is created and connected to the small intestine, bypassing most of the stomach and part of the intestines.	More effective for long-term weight loss (~60–70% excess weight loss). Often resolves type 2 diabetes more effectively, reduces hunger hormones more than a sleeve. May lead to greater metabolic improvements.	Anastomotic leaks (0.4%-2%), postoperative hemorrhage (1.9%-4.4%), small bowel obstruction is most commonly caused by an internal hernia (1%-9%), Anastomotic stricture (2.9%-23%), marginal ulcers, gastrogastric fistula (1.5%-6%), long-term weight regain after 5 and 10 years (10%, 20%).

Discussions

The escalating prevalence of obesity highlights the urgent need for effective, safe, and accessible therapeutic strategies [59]. Bariatric surgery remains among the most efficacious interventions for achieving significant and durable weight reduction. However, it carries notable surgical risks and postoperative complications, including gastric strictures, anastomotic leaks, infections, thromboembolic events, nutritional deficiencies, and frequent reoperations. Furthermore, rapid weight loss following surgery predisposes patients to nutritional deficiencies, GI dysfunction, and eventual weight regain, which compromises long-term success. Access to surgery is also limited by healthcare infrastructure, insurance coverage, and patient eligibility.

In this context, pharmacological therapies, particularly GLP-1RAs, have emerged as transformative alternatives. Agents such as liraglutide and semaglutide consistently demonstrate meaningful weight reduction with favorable safety profiles and greater accessibility compared to surgical approaches. They demonstrate substantial weight loss, but do not fully match the outcomes typically seen with bariatric surgery while offering supplementary benefits for metabolic and cardiovascular health. Similarly, PHEN-TPM has shown robust weight loss outcomes through distinct mechanisms, making it a viable option for patients seeking non-surgical management. Collectively, these medications bridge a critical therapeutic gap for individuals who are either ineligible for or unwilling to undergo invasive interventions. The adverse effects of GLP-1-based therapy rates vary between studies and doses.

## Conclusions

The global obesity epidemic necessitates multifaceted interventions that balance efficacy, safety, and accessibility. This review highlights how pharmacologic agents show promise for obesity treatment by producing meaningful weight loss results and improving metabolic health indicators. The evaluation recognizes the adverse effects that include GI problems and other complications, which need to be studied against the positive outcomes of treatment. Medical professionals should use patient-specific factors, comorbidities, and treatment objectives to determine the appropriate use of these medications. The analysis supports a patient-focused treatment plan that combines behavioral approaches with pharmacological interventions and procedural methods according to each person's requirements. Future research is expected to improve the understanding of obesity pathophysiology and optimize long-term outcomes. Importantly, GLP-1 RAs should not be considered in isolation but as integral components of a comprehensive, patient-centered approach that combines behavioral, pharmacologic, and surgical modalities.

Future directions should prioritize long-term outcome studies, direct comparisons between pharmacologic and surgical therapies, and initiatives to improve affordability and global access. With continued innovation, GLP-1 RAs, dual GIP/GLP-1RA, and triple GIP/GLP-1/glucagon RAs have the potential to redefine the standard of care in obesity treatment and offer a safe, effective, and non-surgical weight loss management option for patients who are reluctant to undergo bariatric surgery or are ineligible due to medical or personal reasons. While bariatric surgery remains the gold standard for durable weight loss, GLP-1RAs bridge the gap between lifestyle interventions and surgical procedures.
